# 

**Published:** 1843-07-22

**Authors:** 


CONTENTS.

A Course of Clinical Lectures, delivered at the Hôtel Dieu, Paris, for the Session 1842–'43. By A. F. Chomel, M.D. Lecture 9, - - - - - - 157

Remarks on the Influenza. By David R. Tucker, M. D. - - - - - 159

*Jefferson Medical College.*

Clinic of Professor Mitchell, - - 161

*Bibliographical Notices.*

1. Medical Clinic: Diseases of the Abdomen. By G. Andral, Professor of the Faculty of Medicine of Paris, &c. &c. Condensed and Translated, with Observations. By D. Spillan, M. D., &c. &c. - - - - - - 162
2. Medical Clinic: Diseases of the Chest. By G. Andral. Condensed and Translated, with Observations. By D. Spillan, M. D., &c. - - - - - 162

The Physical Diagnosis of Diseases of the Lungs. By Walter Hayle Walshe, M. D., Professor of Pathological Anatomy in the University College, London, &c. &c. - - - - - 162

Effects of Climate on Phthisis, - 163

Statistics of Tænia, - - - 163

*Retrospect of the Medical Sciences.*

Case of Strangulated Intestines, from rotation of the Sigmoid Flexure—with Remarks. By J. Bigelow, M. D. - 164

Calumba Root, - - - - - 165

Concise Historical sketch of the Progress of Pharmacy in Great Britain. By Jacob Bell, - - - - - 165

Pyelitis, - - - - - - - 166

Unique Species of Hernia, - - - 167

Case of Total Absence of the Spleen, - 168

Blood corpuscles and spermatozoa of the Camelidæ, - - - - - - 168

Spermatozoa in the fluid of Hydrocele, 168

